# An Analysis of Antibody Response to COVID-19 Vaccination Among Medicos in a Predominantly Tribal State in India: A Comparative Study

**DOI:** 10.7759/cureus.61154

**Published:** 2024-05-27

**Authors:** Kumari Asha Kiran, Sushma Kumari, Usha Saroj, Manisha Kujur, Anit Kujur, Mithilesh Kumar, Smiti Narain, Venkatesh N, Jeseena K

**Affiliations:** 1 Preventive Medicine, Rajendra institute of Medical Sciences, Ranchi, IND; 2 Blood Bank, Rajendra Institute of Medical Sciences, Ranchi, IND; 3 Blood Bank, Rajendra institute of Medical Sciences, Ranchi, IND; 4 Community Medicine, Rajendra Institute of Medical Sciences, Ranchi, IND; 5 Preventive and Social Medicine, Rajendra institute of Medical Sciences, Ranchi, IND

**Keywords:** immunoglobulin g, vaccine, covaxin and covishield, antibody response, sars-cov-2 infection

## Abstract

Introduction

Global health is still being impacted by the coronavirus disease 2019 (COVID-19) pandemic.

Objectives

We evaluated the antibody response in this study in individuals who received two doses of the COVID-19 vaccination, both with and without a history of SARS-CoV-2 infection.

Methodology

It was a hospital-based cross-sectional study conducted among healthcare personnel at a tertiary institution of a predominantly tribal state in India.

Results

A total of 187 medical students made up the vaccinee group; the majority (152; 81.3%) were between the ages of 18 and 23; 128 (68.4%) of the students were female; and 104 (55.6%) had received the Covishield (AstraZeneca plc, England, UK) vaccination. Of the subjects, 51 (27.3%) had a history of COVID-19 infection. For those who were infected, the antibody titer peaked after six months, whereas it took twice as long for those who were not. Up to a year later, the antibody titers for Covaxin (Bharat Biotech, Hyderabad, India) and Covishield remained equal; however, Covishield titers drastically decreased while Covaxin stayed constant when an infection history was present.

Conclusion

The study's findings show that immunization in individuals who have previously contracted COVID-19 induces a higher level of antibody response than immunization in individuals who have not previously contracted the virus.

## Introduction

The coronavirus disease 2019 (COVID-19) pandemic is continuing to affect global health, according to a WHO report. Up until December 2022, 642,924,560 COVID-19 cases were confirmed, resulting in 6,625,029 deaths [1]. The Covishield and Oxford-AstraZeneca vaccines (AstraZeneca plc, England, UK) are produced domestically by the Serum Institute of India (Pune, India), the largest vaccine manufacturer in the world. It is created from an attenuated chimpanzee adenovirus. International clinical investigations of the Covishield vaccination found that, compared to half-dose administration, full-dose administration resulted in an 80% effectiveness rate [2]. Contrarily, Covaxin is a domestically produced vaccine produced by Bharat Biotech with government support. It has been demonstrated that these vaccines, which are given as two intramuscular shots, provide protection by inducing the production of anti-S-protein receptor binding domain (S-RBD) immunoglobulin G (IgG), IgM, and IgA isotypes, with neutralization activity able to prevent RBD from binding to the cognate receptor of angiotensin-converting enzyme 2 (ACE2) [3,4]. These antibodies' levels in sera, especially the neutralizing antibody levels, can be measured to determine the degree of protection brought on by prior infection or COVID-19 immunization [5].

The characteristics, reliability, and longevity of antibody responses in individuals with COVID-19 are subjects of considerable debate [6]. While certain studies have observed a swift decrease in antibody immunity, delayed onset with minimal antibody levels, or even a total absence of enduring antibodies, others have reported consistent and persistent antibody protection [7,8]. Additional research is needed to confirm the safety and efficacy of booster shots, especially in individuals who are immunocompromised or receiving immunosuppressive treatments, to determine the best vaccination schedule and combination strategies. Since the advantages of receiving a COVID-19 immunization substantially outweigh any potential hazards, US public health officials have encouraged the FDA to make a decision regarding booster vaccines [9]. Plans for booster shot programs have been announced by several nations. Elevated levels of infection-blocking "neutralizing" antibodies have resulted from third shots of vaccines developed by Moderna, Pfizer-BioNTech, Oxford-AstraZeneca, and Sinovac. These doses were provided several months after the second dose [10].

Conversely, it has been demonstrated that those who have already contracted COVID-19 [11] have some humoral immunity, although they are still susceptible to reinfection [12,13].

In this study, the antibody-mediated immune response of vaccinated individuals with prior COVID-19 infections was explicitly compared with that of vaccinated individuals without prior COVID-19 infections.

## Materials and methods

The study was a cross-sectional study conducted at tertiary care centers in Ranchi and Jharkhand. The study population was healthcare students of Rajendra Institute of Medical Sciences (RIMS) (undergraduates, nursing, and paramedical). Our inclusion criteria were the students who were willing to participate and who received two doses of both COVID-19 vaccines named Covishield and Covaxin. A convenient sampling method was chosen for sample collection. The data were gathered using a pretested structured Google form that contained fundamental information like demographic profile, vaccination details, and history of infection.

Study procedure

After receiving institutional ethics committee approval (395 IEC, RIMS dated 07-12-2021), the study was carried out. After receiving permission from the nodal authority for that setting, precise documentation of the patient's immunization status was obtained from the hospital's registered COVID-19 vaccination center. We contacted 212 individuals in the vaccination center during our three months of study period from January 2022 to March 2022. Out of 212 individuals, 187 individuals who had given their consent were included in our study. We collect data from participants who are consecutively selected in order of appearance at the time of the interview, based on convenient accessibility. Self-reported vaccination status was checked and confirmed using any available data sources, including vaccination center records and certificates. The COVID-19 vaccination record includes the brand of the shot as well as information about the infection. The students' blood samples were taken in ethylenediaminetetraacetic acid (EDTA) vials for antibody titer after obtaining proper informed consent from them. All of the blood samples were centrifuged at a correct balance for 10 minutes at 10,000 RPM to separate plasma. The Blood Bank used an automated chemiluminescent microparticle immunoassay (CMIA) test for both the qualitative and quantitative detection with the Abbott Architect i1000SR model (Abbott Laboratories, IL, US) by using the SARS CoV-2 IgG II Quant assay reagent (Abbott Laboratories) to test the separated plasma from all the blood samples for anti-SARS CoV-2 IgG antibodies against the spike receptor-binding domain (RBD) of SARS-CoV-2. The manufacturer's instructions state that plasma samples are positive when the IgG levels are greater than 50 AU/ml. Microsoft Excel (Version 13, Microsoft Corporation, Redmond, WA, US) was used to enter the data and create the template. SPSS software version 22 (IBM Corporation, Armonk, NY, US) was used to analyze the data.

## Results

A total of 187 medical students made up the vaccine group; the majority (152; 81.3%) were between the ages of 18 and 23; 128 (68.4%) were female; and 59 (55.6%) had received the Covishield vaccine. Tribals accounted for 47 individuals, constituting 25.1% of the total. A history of COVID infection was present in 51 (27.3%) participants (Table 1). Among them, 12 (23%) were female and 2 (4.3%) were males. Of the total 187 participants, 28 individuals (15%) had a COVID infection over six months ago while 17 (9.1%) had been infected less than a month prior.

**Table 1:** Profile of participants (n=187) The majority (152; 81.3%) were predominantly female and aged between 18 and 23. Out of 187 participants, 104 (55.6%) had received the Covishield vaccination and 51 (27.3%) had a history of past COVID-19 infection.

		Frequency(n)	Percentage (%)
Age	18-23 yrs	152	81.3
	23-28 yrs	23	12.3
	>28 yrs	12	6.4
Gender	Male	59	31.6
	Female	128	68.4
Ethnicity	Non-tribal	140	74.9
	Tribal	47	25.1
Presence of co-morbidity	Present	9	4.8
	Absent	131	70.1
	Do not know	47	25.1
H/o.covid infection	Yes	51	27.3
	No	136	72.7
Time since the last infection	<1 month	17	9.1
	1-6 month	6	3.2
	>6 month	28	15.0
H/o vaccination	yes	187	100
Type of vaccine taken	Covaxin	83	44.4
	Covishield	104	55.6
Total(n)		187	100

**Figure 1:**
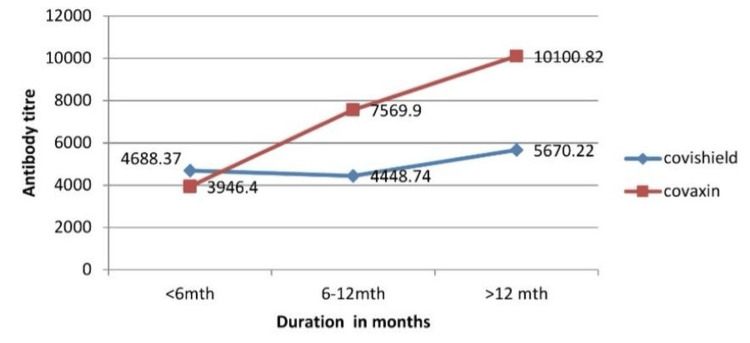
Mean antibody titer (AU/ml) in vaccinated (without h/o. COVID infection) Mean antibody titer was higher in participants vaccinated with Covaxin

Among the 136 participants with no history of COVID-19 infection, 81 (59.55%) had received the Covishield vaccine while 55 (40.45%) received Covaxin. The mean antibody titer after 12 months was 10100.82 AU/ml for Covaxin, approximately half of that for Covishield. Initially, the antibody titer for Covaxin was lower than that for Covishield. However, after six months, the antibody titer for Covaxin began to increase more rapidly than that for Covishield, maintaining a higher level throughout the duration of the study(Figure 1).

**Figure 2:**
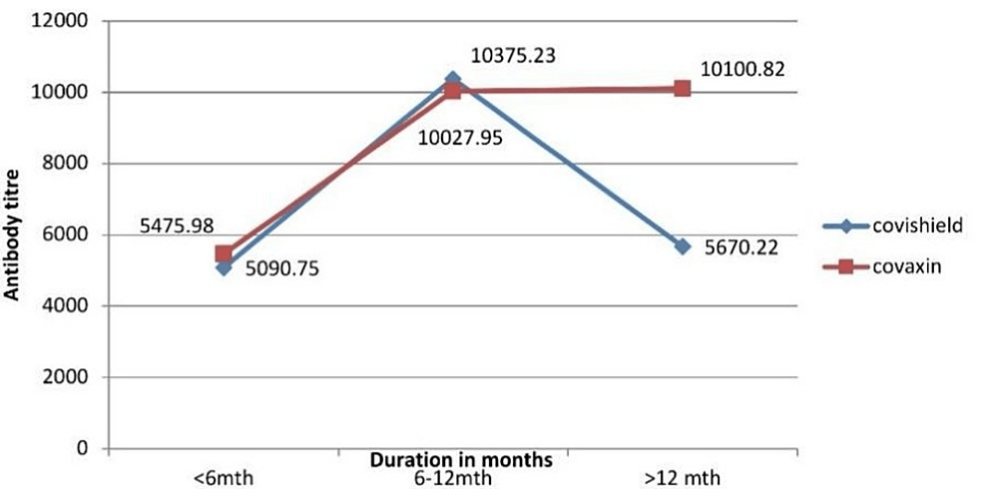
Mean antibody titer in the vaccinated (with H/O COVID infection) Covaxin and Covishield titers were similar till 12 months; after that, the Covishield titer declined sharply.

In participants with a history of COVID infection, the mean antibody titer remained comparable between Covaxin and Covishield for the first six months. Subsequently, there was a sharp decline in the antibody titer for Covishield, whereas the titer for Covaxin remained stable. Even after 12 months, the antibody titer in Covaxin exceeded 10,000 AU/ml, whereas the titer for Covishield declined to baseline levels (Figure 2). The antibody titer peaked in six months for infected individuals and took twice as long for non-infected individuals (Figures 1, 2).

**Figure 3:**
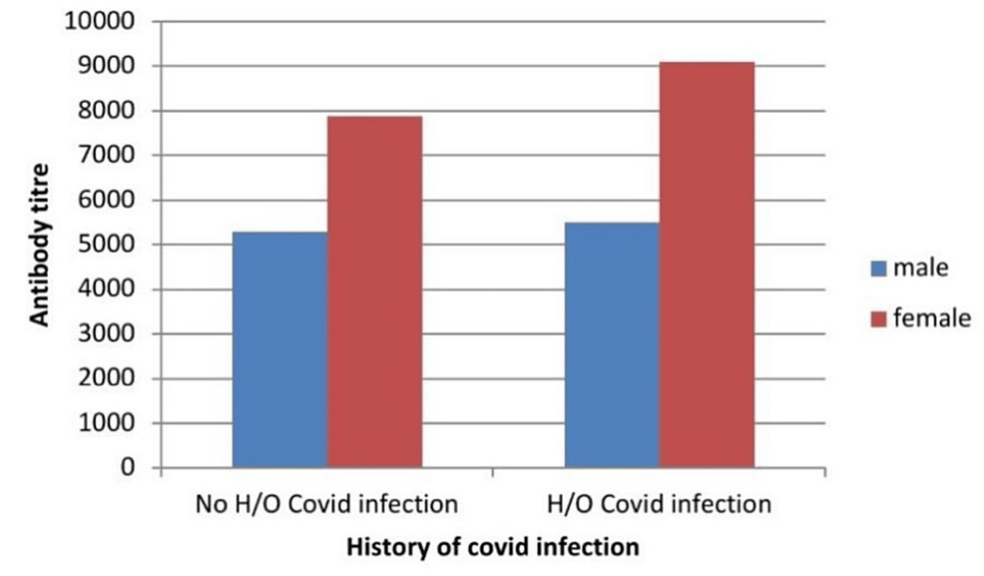
Gender-wise distribution of the mean antibody titer (AU/ml) Females were more in number in both the infected and non-infected groups.

Upon gender-based analysis, we observed that the mean antibody titer was higher among females in both the infected and non-infected groups (Figure 3).

## Discussion

A global immunization effort capable of delivering safe and effective vaccinations is undoubtedly the best way to combat the COVID-19 pandemic. When COVID-19 vaccinations began in India in 2021, frontline workers and high-risk groups, like the elderly and immunocompromised, were given priority. Whereas Covishield showed an efficacy of around 90%, Covaxin's efficacy is just about 80% [14]. In this study, we investigated the impact of a prior COVID-19 infection on SARS-CoV-2 antibodies in all subjects who received both doses of the immunization. Protection following vaccination depends on a coordinated response by multiple immune systems, collectively giving rise to durable immunity.

This study's key observation was that fully vaccinated people who had previously contracted COVID-19 had much greater antibody levels than fully vaccinated people who had never contracted the virus. This was similar to the finding in the study of Ali Hamad et al. [15]. It was shown that those with a history of infection had a quicker peak in antibody levels; however, their drop in levels was faster than that of non-infected individuals. A similar finding was reported by previous studies [16,17]. The high antibody levels in previously infected groups are most likely the result of the multiplication of B cells and the production of antibodies after both the infection and the vaccine. Although the vaccines work by stimulating the immune system to respond in the same way it would after a virus infection, they contain slightly different conformations of the virus protein [18]. The faster reduction in antibodies among non-infected persons found in their study was in contrast to the faster decline in infected individuals observed in our study [14]. The variation in vaccinations could be the cause. The vaccines employed in our study were Covishield and Covaxin while BNT162b2 (Pfizer-BioNTech) was used in their study. The reason behind this could be because mRNA vaccines effectively initiate B cell responses and secrete antibodies against SARS-CoV-2, making them more effective [17]. Further lipid nanoparticle-based mRNA delivery for the BNT162b2 vaccine could also introduce antigens in a different manner to the immune system than in a real viral infection, which could lead to differential antigen kinetics and antibody generation [16,19]. In both the infected and non-infected groups, we discovered that the mean antibody titer in females was higher than in males. This was similar to the findings observed by other studies [20,21]. The higher antibody response to vaccinations in this group can be explained by the fact that females are known to mount stronger and faster innate and adaptive immune responses than males [22]. The antibody titers of both vaccines in infected participants of our study were similar, but after a year, the Covishield titer sharply declined, while the Covaxin titer stayed unchanged. This may be the case because Covaxin is used in combination with immune stimulants, sometimes referred to as vaccine adjuvants (e.g., Alhydroxiquim-II), to boost immunity and generate immunity that lasts longer [20].

Limitation of the study

Our study was observational, so a longitudinal study will be required to confirm this finding if and when a booster dose becomes necessary.

## Conclusions

The results of this study demonstrate that immunization with a history of COVID-19 infection elicits a larger antibody response than immunization without a history of infection. However, a double dose of vaccine is now insufficient for persons who have already contracted the infection, especially with Covishield. These results can be useful in developing vaccination policy initiatives.
